# Self-Administration of Right Vagus Nerve Stimulation Activates Midbrain Dopaminergic Nuclei

**DOI:** 10.3389/fnins.2021.782786

**Published:** 2021-12-16

**Authors:** Jackson Brougher, Umaymah Aziz, Nikitha Adari, Muskaan Chaturvedi, Aryela Jules, Iqra Shah, Saba Syed, Catherine A. Thorn

**Affiliations:** Department of Neuroscience, University of Texas at Dallas, Richardson, TX, United States

**Keywords:** VNS (vagus nerve stimulation), lateralization, dopamine, self-administration, ventral tegmental area, substantia nigra, neural stimulation, c-fos

## Abstract

**Background:** Left cervical vagus nerve stimulation (l-VNS) is an FDA-approved treatment for neurological disorders including epilepsy, major depressive disorder, and stroke, and l-VNS is increasingly under investigation for a range of other neurological indications. Traditional l-VNS is thought to induce therapeutic neuroplasticity in part through the coordinated activation of multiple broadly projecting neuromodulatory systems in the brain. Recently, it has been reported that striking lateralization exists in the anatomical and functional connectivity between the vagus nerves and the dopaminergic midbrain. These emerging findings suggest that VNS-driven activation of this important plasticity-promoting neuromodulatory system may be preferentially driven by targeting the right, rather than the left, cervical nerve.

**Objective:** To compare the effects of right cervical VNS (r-VNS) vs. traditional l-VNS on self-administration behavior and midbrain dopaminergic activation in rats.

**Methods:** Rats were implanted with a stimulating cuff electrode targeting either the right or left cervical vagus nerve. After surgical recovery, rats underwent a VNS self-administration assay in which lever pressing was paired with r-VNS or l-VNS delivery. Self-administration was followed by extinction, cue-only reinstatement, and stimulation reinstatement sessions. Rats were sacrificed 90 min after completion of behavioral training, and brains were removed for immunohistochemical analysis of c-Fos expression in the dopaminergic ventral tegmental area (VTA) and substantia nigra pars compacta (SNc), as well as in the noradrenergic locus coeruleus (LC).

**Results:** Rats in the r-VNS cohort performed significantly more lever presses throughout self-administration and reinstatement sessions than did rats in the l-VNS cohort. Moreover, this appetitive behavioral responding was associated with significantly greater c-Fos expression among neuronal populations within the VTA, SNc, and LC. Differential c-Fos expression following r-VNS vs. l-VNS was particularly prominent within dopaminergic midbrain neurons.

**Conclusion:** Our results support the existence of strong lateralization within vagal-mesencephalic signaling pathways, and suggest that VNS targeted to the right, rather than left, cervical nerve preferentially activates the midbrain dopaminergic system. These findings raise the possibility that r-VNS could provide a promising strategy for enhancing dopamine-dependent neuroplasticity, opening broad avenues for future research into the efficacy and safety of r-VNS in the treatment of neurological disease.

## Introduction

Stimulation of the left cervical vagus nerve (l-VNS) is an FDA-approved therapeutic approach for a wide range of neurological diseases, including epilepsy, major depressive disorder, migraine, and stroke. Moreover, recent research is rapidly expanding the clinical indications for which stimulation of the “wandering” vagus nerve may provide therapeutic benefit. Cervical VNS is currently under investigation for multiple other neurological and neurodegenerative disorders, including Alzheimer’s disease (Chang et al., 2018; Slater and Wang, 2021), trauma and anxiety disorders (Marin et al., 2014; Noble et al., 2019), and autism (Engineer et al., 2017; van Hoorn et al., 2019), among others (Hays, 2016; Wang et al., 2021). Though the mechanisms of l-VNS efficacy are incompletely understood, it has been shown that VNS exerts wide-ranging neurological effects in part through activation of the broadly projecting nucleus of the solitary tract and several downstream neuromodulatory nuclei, which include the noradrenergic locus coeruleus (LC), the serotonergic raphe nuclei, and the cholinergic medial forebrain (Detari et al., 1983; Krahl et al., 1998; Dorr and Debonnel, 2006; Osharina et al., 2006; Cunningham et al., 2008; Manta et al., 2009; Ruffoli et al., 2011; Collins et al., 2021). Coordinated activation of these neuromodulatory systems is thought to promote therapeutic neuroplasticity, resulting in improved clinical outcomes (Hays et al., 2013; Conway and Xiong, 2018; Wang et al., 2021).

Midbrain dopaminergic signaling is widely recognized to play a key role in promoting reward-related neuroplasticity throughout the brain (Schultz, 1998; Baik, 2013; Volkow et al., 2017; Speranza et al., 2021), though the role of dopamine in VNS efficacy is less well-studied. Recently, Han et al. (2018) reported a remarkable lateralization in the anatomical and functional connectivity between the vagus nerves and midbrain dopaminergic nuclei. Specifically, these authors demonstrated that optogenetic stimulation of selectively targeted, gut-innervating vagal neurons located in the right, but not left, nodose ganglion (NG) resulted in strong activation of the ventral tegmental area (VTA) and substantia nigra pars compacta (SNc). Moreover, right, but not left, NG stimulation was sufficient to induce striatal dopamine release and appetitive behavioral responses. The clinical implications of these recent findings for the further development of therapeutic VNS remain unclear. VNS-mediated manipulation of the dopamine system could offer powerful additional neuroplasticity-promoting mechanisms by which to achieve therapeutic effects. However, it is unknown whether stimulation of the right vagus nerve (r-VNS) using traditional non-selective electrical stimulation of the cervical fibers would be sufficient to activate the midbrain dopamine system. Nor is it clear whether traditional cervical VNS produces differential dopaminergic activation when applied to the right vs. the left cervical nerves.

In the current study, we compare the appetitive behavioral effects of r-VNS and l-VNS in rats, and ask whether lateralized stimulation produces differential activation of neurons within midbrain dopaminergic nuclei. We first tested the appetitive effects of r-VNS and l-VNS using a VNS self-administration assay. After completion of the behavioral task, animals were sacrificed and c-Fos expression within the expression within the VTA and SNc was quantified to examine neuronal activation in these regions following r-VNS vs. l-VNS. Our behavioral and histological results are consistent with a striking lateralization of vagal-mesencephalic signaling, and suggest that standard electrical stimulation of the right cervical vagus nerve is capable of producing strong activation of the midbrain dopaminergic system.

## Materials and Methods

All procedures were approved by the University of Texas at Dallas Institutional Animal Care and Use Committee and are in accordance with the National Institutes of Health guide for the care and use of laboratory animals.

### Animal Subjects

Fourteen adult female Long-Evans rats, aged 8–14 weeks at study start, were used in these experiments. Rats were housed in a 12:12 h reverse light cycle room with *ad libitum* access to water (lights on: 6:00 pm) and all handling and training occurred during their active cycle. Prior to cuff implantation surgery, rats were handled for at least 3 daily 15-min habituation sessions.

### Vagus Nerve Cuff Electrode Implantation

At study start, rats were randomly assigned to l-VNS (*n* = 7) or r-VNS (*n* = 7) treatment groups. Vagus nerve cuff electrodes consisted of platinum-iridium leads (Sigmund Cohn, #10IR9/4T) ensheathed in MicroRenathane tubing (Braintree Scientific, #MRE080), and were assembled in-house according to published methods (Sanchez et al., 2020). The stimulating cuff electrode was implanted around the targeted cervical vagus nerve as previously described (Porter et al., 2012; Tseng et al., 2020). Briefly, an incision was made 1 cm from the midline on either the right or left side, and the targeted cervical vagus nerve was bluntly dissected from the carotid artery and placed inside the cuff. A second incision was then made on the midline of the skull at the occipital and parietal bones, and cuff electrode leads were tunneled subcutaneously, exited through this second incision, and attached to a headcap/connector (Omnetics, #A24002-004). Cuff function was validated during surgery, for both left- and right-side implants, by delivering a single 10-s train of electrical stimulation (amplitude = 0.8 mA, pulse frequency = 30 Hz, pulse width = 100 μs biphasic) using an isolated pulse stimulator (A-M Systems, Model 2100) to evoke a brief cessation of breathing consistent with the Hering-Breuer reflex (Bucksot et al., 2020). Following cuff validation, the neck incision was sutured. Fascia was cleared from the skull, the headcap was secured with bone screws and dental cement, and the cranial incision closed with sutures. Rats were given a 1-week surgical recovery period prior to the start of behavioral training. For 3 days post-surgery, rats were administered Baytril (enrofloxacin, 0.5 mg/5 g) and Rimadyl (carprofen, 2 mg/5 g) tablets (Bio-Serv, Flemington, NJ, United States).

### Vagus Nerve Stimulation Self-Administration

After recovery from surgery, rats underwent the VNS self-administration assay, which included Acclimation, VNS Self-Administration (VNS-SA), Extinction (EXT), and Reinstatement (R) stages. Throughout the duration of the training protocol, beginning 24 h prior to the start of Acclimation, rats were lightly food restricted. Subjects received 5 pellets of rat chow (ca. 14–18 gm; Labdiet Prolab RMH 1800) each day, delivered in the homecage immediately following the training session. Weights were monitored daily prior to feeding to ensure animals maintained at least 90% of their free-feeding weight throughout the study.

During Acclimation, rats were placed in a MotoTrak training booth (30 cm × 13 cm × 25 cm booth; Vulintus, Inc., Louisville, CO) overnight (8–12 h) and trained to press a lever (>1.5 degree deflection from horizontal) extending 1 cm inside the booth to receive a 45 mg food pellet (Bio-Serv, Flemington, NJ; #F0021). A 2-s time-out period followed each pellet delivery before a subsequent trial could be initiated. Rats were required to perform at least 100 rewarded presses within a single overnight Acclimation session before beginning VNS-SA. If a subject failed to perform at least 100 presses, they were given a 24-h rest period before receiving an additional acclimation session. All rats completed the Acclimation stage in 1–4 sessions (mean = 2.4).

**FIGURE 1:**
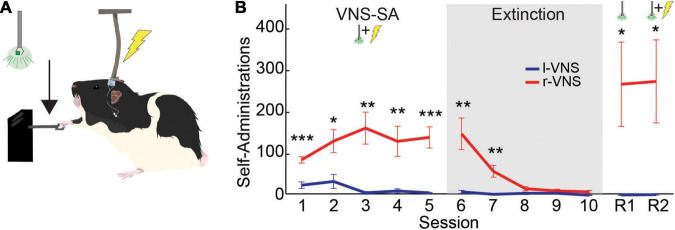
Rats self-administer r-VNS but not l-VNS. **(A)** During VNS-SA sessions, r-VNS or l-VNS was delivered along with a visual cue (488 nm LED) immediately upon detection of a lever deflection. **(B)** Lever-press performance of r-VNS (red; *n* = 7) and l-VNS (blue; *n* = 7) cohorts throughout the self-administration assay. Training stages included VNS self-administration (VNS-SA), Extinction, cue-only reinstatement (R1), and yoked-stimulation reinstatement (R2) sessions. Student’s *t*-tests were used to test for between-group differences in behavioral performance within each training session and corrected for multiple comparisons using false discovery rate. For sessions in which FDR-corrected comparisons indicated the presence of a statistically significant between-group difference, uncorrected *p*-values are denoted: *p < 0.05, **p < 0.01, ***p < 0.001.

Following Acclimation, rats underwent five 2-h VNS-SA sessions (1/day) in which food pellets were removed and pressing behavior was instead paired with VNS delivery and the onset of a visual cue (Figure 1A). VNS stimulation parameters were identical to those shown in our previous studies of l-VNS to induce neuroplasticity within the motor cortex (Tseng et al., 2020; Brougher et al., 2021). Immediately upon detection of a lever press, a single 0.5 s train of 16 pulses (amplitude = 0.8 mA, pulse frequency = 30 Hz, pulse width = 100 μs biphasic) was delivered through the implanted cuff electrode. The same stimulation parameters were used for both l-VNS and r-VNS groups. The visual cue consisted of a green (488 nm) LED located outside of the booth directly above the lever. Visual cue onset was simultaneous with and for the same duration as the VNS train (0.5 s).

Following VNS-SA, rats received five 2-h EXT sessions (1/day). During EXT, the VNS stimulator and LED remained off, and lever presses no longer resulted in stimulation or visual cue delivery.

After EXT, animals underwent a single 2-h session of visual cue-only reinstatement (R1). During R1, successful lever presses resulted in the presentation of the visual cue only, but no VNS was delivered.

Following R1, subjects underwent a second 2-h cue + VNS reinstatement session (R2). During R2, r-VNS rats received delivery of both the visual cue and VNS immediately upon detection of each lever press, as they did during VNS-SA sessions. To ensure both r-VNS and l-VNS treatment groups received equal amounts of stimulation during this final R2 session, stimulation of each rat in the l-VNS group was yoked to that of an r-VNS subject. Yoked r-VNS and l-VNS rats were run simultaneously and both received cue and VNS delivery contingent on the lever pressing behavior of the r-VNS rat. Ninety minutes after the completion of R2, rats were sacrificed for histological analyses.

### c-Fos Immunohistochemistry

Ninety minutes after the final reinstatement session, rats were deeply anesthetized with sodium pentobarbital/phenytoin (150/50 mg/kg, i.p.) and transcardially perfused with ice-cold phosphate buffered saline (PBS), followed by 4% paraformaldehyde in PBS. Brains were removed and stored in 4% paraformaldehyde overnight for fixation. The following day, brains were transferred to a 30% sucrose solution for cryoprotection.

Three subjects in each group were randomly chosen for inclusion in the histological analyses. A cryostat was used to make brain slices through the VTA/SNc (AP: −5.2 to −5.3 mm from Bregma) and the LC (−9.6– to −9.7 mm from Bregma) at 20 μm thickness. Slices were washed (3X in PBS), followed by 30 min permeabilization with 0.5% Triton-X in PBS. Slices were again washed and blocked for 1 h in 2.0% BSA in PBS. Slices were then washed and incubated overnight at 4°C in a primary antibody cocktail to label tyrosine hydroxylase (TH) and c-Fos (chicken anti-TH, 1:1,000 dilution, Abcam #ab76442; mouse anti-c-fos, 1:1,000, Abcam #ab208942). The following day, slices were washed and incubated at room temperature for 1 h in secondary antibody solution (anti-chicken IgY conjugated to Alexa Fluor 555, 1:1,000 dilution, Abcam #ab150170; anti-mouse IgG conjugated to Alexa Fluor 488, 1:1,000 dilution, ThermoFisher #A28175). Finally, slices were washed and mounted on slides in a DAPI containing mounting medium (DAPI Fluoromount-G, SouthernBiotech #0100-20).

For each subject examined, three alternating slices were imaged per nucleus of interest. VTA, SNc, and LC in both left and right hemispheres were imaged for subsequent Mean Gray Value (MGV) and cell counting analyses. Images were made using an Olympus BX51 fluorescent microscope. Images for MGV were taken at 10x magnification; images for cell counting were taken at 20x magnification.

MGV quantification was performed bilaterally in each analyzed slice imaged at 10x. For each hemisphere and nucleus of interest, regions of interest (ROIs) were drawn by hand in ImageJ using TH+ fluorescence as an indicator for nucleus boundaries based on prior literature (Boekhoudt et al., 2017; Farrand et al., 2020). For each image, ROIs were accepted for analysis if detected TH fluorescence (raw integrated density) within the ROI made up at least 95% of the total TH fluorescence within the image. In images containing multiple TH+ nuclei (i.e., VTA and SNc), sections of the adjacent, non-target nuclei were cropped prior to ROI validation. Midline was determined in VTA images by the presence of the periaqueductal gray dorsal to the VTA. For each ROI, overall c-Fos expression was quantified in ImageJ as the MGV of c-Fos immunofluorescence within the ROI. MGVs were then averaged across the 3 slices per nucleus, to obtain a measure of nucleus- and hemisphere-specific c-Fos expression for each rat.

Specific cell counts were also obtained for each nucleus of interest using additional images taken at 20x magnification. Images were centered on the densest population of TH+ cells within each nucleus. As for MGV, ROIs were drawn in ImageJ using the boundaries of TH expression to define nucleus boundaries, and ROIs were accepted for analysis if 95% of the TH+ signal in the image was contained within the ROI. Images were manually quantified to obtain specific cell counts for DAPI+, TH+, and/or c-Fos+ cells. Images were pseudocolored in ImageJ for quantification (TH = red, c-Fos = green, DAPI = blue). Each ROI was quantified by 2 graders, both blinded to the subject’s treatment condition and the other grader’s counts. Only DAPI+ cells within the plane of focus were counted; cells were classified into 1 of 4 categories: (1) DAPI+ only, (2) TH+ and DAPI+, (3) c-Fos+ and DAPI+, or (4) c-Fos+ and TH+ and DAPI+. For each grader and nucleus of interest, percentages of (1) TH+ cells, (2) c-Fos+ cells within the TH+ population, and (3) c-Fos+ cells within the TH− population were calculated and averaged across relevant ROIs in all three slices per nucleus, and then averaged across the 2 hemispheres to obtain average count values for each cell type in each nucleus. Percentages were then averaged across the two graders to obtain cell-type-specific quantification of c-Fos expression within each nucleus for each rat.

### Data Analysis

Behavioral data (lever presses per session) were analyzed in R 4.0.3 (R Core Team, 2021) using a two-way mixed ANOVA, with treatment group as a between-subject factor and session number as a within-subject factor. As Mauchly’s test indicated a lack of sphericity (*p* < 0.0001), Greenhouse-Geisser corrected within factor results are reported. *Post hoc t*-tests were then used to compare lever pressing between l-VNS and r-VNS treatment groups within each session, and corrected for multiple comparisons using false discovery rate (FDR). For all behavioral analyses, statistical significance is reported for FDR-adjusted *p* < 0.05. All summary statistics are reported as mean ± SEM.

For histological MGV analysis, 2-way ANOVA was used to test for differences in c-Fos expression across brain hemispheres and VNS treatment groups. For each nucleus of interest, two-way ANOVAs were followed by Tukey *post hoc* comparisons of c-Fos expression across all four (brain hemisphere × stimulation side) contingencies. Significant differences are reported for *p* < 0.05.

For histological cell counts, the percentages of TH+, c-Fos+/TH+, and c-Fos+/TH− cells within each nucleus of interest were compared between l-VNS and r-VNS treated rats using unpaired Student’s *t*-tests, which were corrected for multiple comparisons using false discovery rate. Significant differences are reported for FDR-adjusted *p* < 0.05.

## Results

Fourteen female rats were implanted with stimulating cuff electrodes around the right (r-VNS: *n* = 7) or left (l-VNS: *n* = 7) cervical vagus nerve. After surgical recovery, all rats were habituated to the lever press task using food rewards during 1–4 overnight sessions prior to the start of VNS-SA (see section “Materials and Methods”). Rats in r-VNS and l-VNS groups performed similarly during habituation (Lever presses during final habituation session: r-VNS: 144 ± 15.66, l-VNS = 137 ± 13.63; *p* = 0.742, unpaired *t*-test). During daily 2-h VNS-SA sessions, each lever press was paired with simultaneous onset of a visual stimulus and delivery of a brief train of VNS (Figure 1A). VNS stimulation parameters were matched in r-VNS and l-VNS treatment groups, and identical to those used in prior studies (Tseng et al., 2020; Brougher et al., 2021).

### Rats Self-Administer Right Cervical Vagus Nerve Stimulation but Not Left Cervical Vagus Nerve Stimulation

During VNS-SA, rats in the r-VNS treatment group quickly began to lever press at high rates to self-administer vagal stimulation, while l-VNS failed to drive similar levels of lever responding [Greenhouse-Geisser corrected 2-way mixed ANOVA, group effect: *F*_(1, 12)_ = 21.528, *p* = 5.7e-4; session effect: *F*_(1.56, 18.7)_ = 4.487, *p* = 0.033; interaction: *F*_(1.56, 18.7)_ = 4.602, *p* = 0.031]. Lever press performance in the r-VNS and l-VNS treatment groups began to significantly diverge during the first VNS-SA session (SA1), ca. 65 min into the 2-h session (Supplementary Figure 1). In each of the 5 VNS-SA sessions, rats that received r-VNS performed significantly more presses per session than rats that received traditional l-VNS (Figure 1B and Table 1). Rats in the r-VNS group increased their rates of lever pressing throughout VNS-SA sessions (r-VNS group, SA1 vs. SA5: *p* = 0.038, paired *t*-test), and performed over 120 presses on average in SA sessions 2 through 5 (Table 1). By contrast, rats that received l-VNS at matched stimulation parameters performed fewer than 40 presses per session (Table 1), and decreased their response rate across VNS-SA sessions (l-VNS group, SA1 vs. SA5: *p* = 0.047, paired *t*-test). Taken together, these results suggest that r-VNS, but not l-VNS, was highly behaviorally reinforcing.

**TABLE 1:** Comparison of lever pressing performance for l-VNS vs. r-VNS treated rats throughout self-administration, extinction, and reinstatement sessions.

	l-VNS	r-VNS	*t*-test

Session	Mean (SEM)	Mean (SEM)	*p*-value (FDR *q*-value)
**VNS self-administration**			
SA1	25.86 (7.91)	86.71 (7.70)	**0.0001** (0.001)
SA2	34.71 (16.66)	131.71 (27.62)	**0.0109** (0.019)
SA3	7.43 (3.43)	163.00 (38.38)	**0.0016** (0.007)
SA4	11.86 (5.35)	131.29 (36.16)	**0.0067** (0.014)
SA5	7.43 (1.02)	140.86 (25.40)	**0.0002** (0.001)
**Extinction**			
EXT1	9.71 (3.62)	149.43 (37.85)	**0.0032** (0.008)
EXT2	3.86 (1.63)	59.14 (14.12)	**0.0022** (0.007)
EXT3	6.29 (2.96)	17.43 (4.72)	0.0684 (0.075)
EXT4	6.43 (1.90)	12.00 (4.33)	0.2617 (0.262)
EXT5	1.71 (0.47)	9.71 (3.68)	0.0518 (0.062)
**Reinstatement**			
R1	2.14 (1.18)	268.57 (101.26)	**0.0219** (0.029)
R2	3.14 (1.10)	275.29 (99.83)	**0.0184** (0.028)

*Student’s t-tests were used to compare lever pressing between treatment groups during each training session and corrected for multiple comparisons using false discovery rate (FDR). Bold denotes a statistically significant difference in behavioral performance between groups for FDR-adjusted q < 0.05.*

Following VNS-SA sessions, rats underwent 5 days of extinction training in which the visual cue and VNS were no longer delivered upon detection of a lever press. During extinction, r-VNS treated rats significantly decreased their lever pressing (r-VNS group, SA5 vs. EXT5: *p* = 0.002), and lever responding in the l-VNS treatment group further declined (l-VNS group, SA5 vs. EXT5: *p* = 0.0003, paired *t*-test). Rats in the r-VNS group continued to press significantly more than those in the l-VNS group during the first two extinction sessions, but response rates were similarly low in both groups during the final three sessions of extinction (Figure 1B and Table 1).

Following extinction, rats underwent two sessions of reinstatement. During the first reinstatement session (R1), the visual cue alone was presented upon lever pressing, but no VNS was delivered. Rats in the l-VNS group continued to press the lever at very low rates during cue-only reinstatement. By contrast, rats in the r-VNS group resumed high levels of lever responding during R1 (Figure 1B and Table 1), suggesting that the visual stimulus itself had acquired strong appetitive value during r-VNS and was sufficient to reinforce lever responding.

During the second reinstatement session on the following day (R2), both visual cue and VNS were delivered. To ensure that l-VNS and r-VNS treatment groups received equal amounts of stimulation during R2, stimulation of each l-VNS rat was yoked to the performance of a rat in the r-VNS treatment group. In R2, as in R1, r-VNS treated rats continued to press the lever at high rates, whereas l-VNS rats continued to exhibit low levels of lever engagement (Figure 1B and Table 1).

Taken together, our results demonstrate that rats will readily self-administer brief bursts of 30 Hz r-VNS, but that l-VNS delivered at equivalent stimulation parameters does not produce similar appetitive behavioral responses. Extensive literature details the importance of dopaminergic signaling in the reinforcement of self-administration behaviors, including during acquisition, extinction, and reinstatement (Olds and Milner, 1954; German and Bowden, 1974; Volkow et al., 2017; Namba et al., 2018; Pitchers et al., 2018; Salinas-Hernández et al., 2018; Wise and Robble, 2020). Our behavioral findings reveal a striking laterality in the reinforcing effects of cervical vagus nerve stimulation, and are consistent with strong activation of the midbrain dopaminergic reward nuclei by r-VNS, but not l-VNS.

### Right Cervical Vagus Nerve Stimulation Self-Administration Enhances c-Fos Expression in Dopaminergic and Noradrenergic Nuclei

To specifically test whether r-VNS self-administration engages midbrain dopaminergic nuclei, we examined c-Fos expression in the VTA and SNc of our rats following the completion of the self-administration assay. As l-VNS efficacy has been previously shown to depend on noradrenergic signaling (Krahl et al., 1998; Furmaga et al., 2011; Grimonprez et al., 2015; Hulsey et al., 2019), we additionally asked whether r-VNS and l-VNS produced similar levels of LC activation.

**FIGURE 2:**
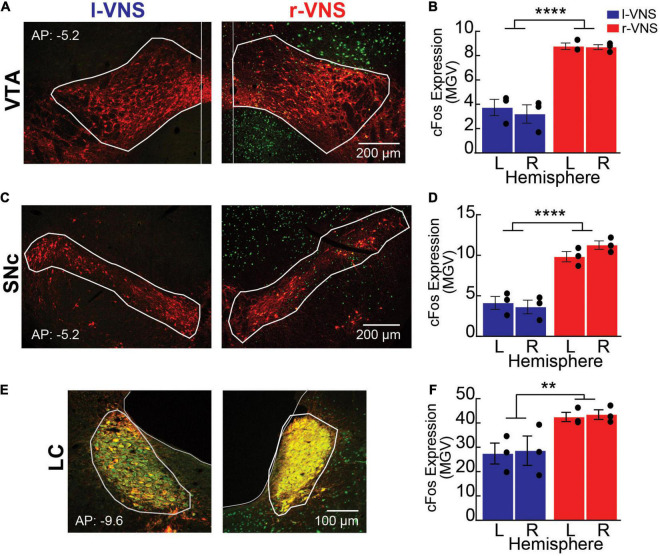
Compared to l-VNS, r-VNS self-administration increases c-Fos expression within catecholaminergic nuclei. **(A,C,E)** Representative 10x images and ROI boundaries used to quantify c-Fos expression within the VTA **(A)**, SNc **(C)**, and LC **(E)** following either l-VNS (left) or r-VNS (right). Sections were stained for tyrosine hydroxylase (red) to label catecholaminergic neurons, c-Fos (green) as a marker of neuronal activation, and the nuclear marker DAPI (omitted for clarity). **(B,D,F)** Mean gray value (MGV) of c-Fos fluorescence was significantly greater following r-VNS self-administration (red) than in l-VNS treated rats (blue) within both left (L) and right (R) brain hemispheres of the VTA **(B)**, SNc **(D)**, and LC **(F)**. **p < 0.01; ****p < 0.0001; 2-way ANOVA between-group comparisons. Within each treatment group, no significant difference in c-Fos expression was observed between left and right brain hemispheres; full statistical results are presented in Tables 2, 3.

For these analyses, rats were sacrificed 90 min after the conclusion of the R2 reinstatement session, and c-Fos expression was examined in the VTA, SNc, and LC bilaterally. Sections were co-stained for TH to identify the boundaries of each nucleus of interest (Figures 2A,C,E). We first compared total c-Fos expression between hemispheres and between l-VNS and r-VNS treated subjects by computing the mean gray value (MGV) within regions of interest (ROIs) defining the VTA, SNc, and LC (Figures 2B,D,F). For all 3 catecholaminergic nuclei, 2-way ANOVAs revealed a significant main effect of stimulation side on c-Fos expression, but no effect of brain hemisphere or interaction effects (Table 2). Tukey *post hoc* comparisons confirmed that r-VNS self-administration resulted in significantly greater c-Fos expression than l-VNS, and this effect was seen in both the VTA and SNc dopaminergic nuclei, as well as in the noradrenergic LC (Table 3). Combined, these results suggest that, compared to l-VNS administration, activation of midbrain dopaminergic “reward” circuits is strongly enhanced following r-VNS self-administration.

**TABLE 2:** Comparison of c-Fos labeling intensity (mean gray value) in left (LH) vs. right (RH) brain hemispheres (hemi) following l-VNS vs. r-VNS treatment (vns_side), for ventral tegmental area (VTA), substantia nigra pars compacta (SNc), and locus coeruleus (LC).

	l-VNS		r-VNS		2-way ANOVA		
	LH	RH	LH	RH			
	
*Nucleus*	Mean (SEM)				p_*vns_side*_ [F_*vns_side*_]	p_*hemi*_ [F_*hemi*_]	p_*int*_ [F_*int*_]
*VTA*	3.73 (0.7)	3.20 (0.8)	8.77 (0.3)	8.70 (0.2)	**0.000 [98.46]**	0.575 [0.34]	0.658 [0.21]
*SNc*	4.13 (0.8)	3.63 (0.8)	9.83 (0.6)	11.27 (0.52)	**0.000 [86.7]**	0.540 [0.41]	0.214 [1.82]
*LC*	27.43 (4.3)	27.3 (4.9)	42.47 (1.9)	43.47 (2.0)	**0.002 [19.6]**	0.894 [0.02]	0.875 [0.03]

*Bold denotes a statistically significant effect for p < 0.05.*

**TABLE 3:** Tukey *post hoc* comparisons of c-Fos labeling intensity (mean gray value) between left (LH) vs. right (RH) brain hemispheres and r-VNS vs. l-VNS treatments for VTA, SNc, and LC.

	RH	LH	LH vs. RH comparisons		r-VNS | RH vs. l-VNS | LH	r-VNS | LH vs. l-VNS | RH
			
	r- vs. l-VNS comparisons		r-VNS	l-VNS		
VTA	**0.0022**	**0.0003**	0.5289	0.9564	**0.0013**	**0.0005**
SNc	**0.0007**	**0.0004**	0.9997	0.8790	**0.0003**	**0.0008**
LC	*0.0651*	**0.0471**	0.9964	1.000	*0.0635*	0.1483

*Bold denotes statistically significant differences in c-Fos intensity for p < 0.05; italics denotes trend toward statistical significance for p < 0.1.*

### Right Cervical Vagus Nerve Stimulation Enhances c-Fos Expression in Dopaminergic and Non-dopaminergic Midbrain Neurons

**FIGURE 3:**
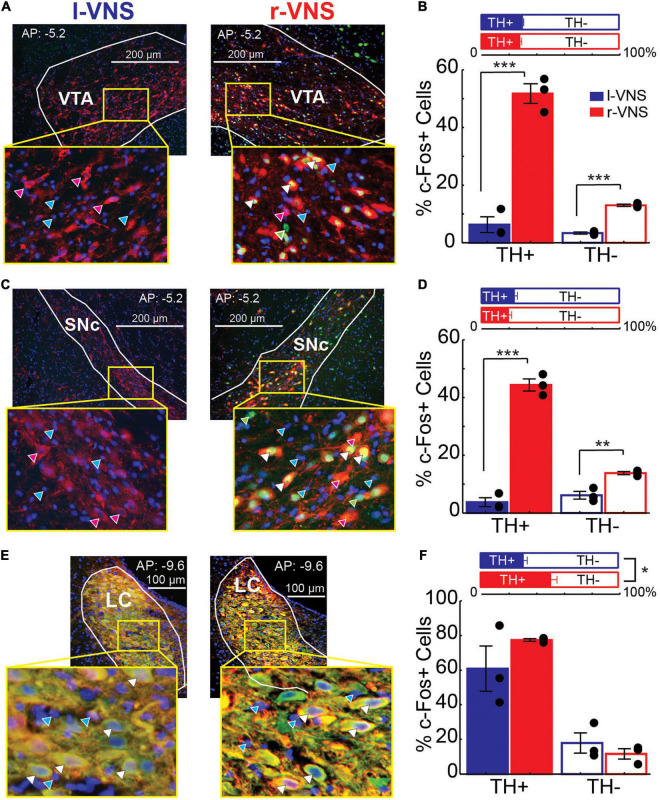
Compared to l-VNS, r-VNS self-administration significantly increases c-Fos expression in both TH+ and TH− cells within catecholaminergic nuclei. **(A,C,E)** Representative 20x images and ROI boundaries used to quantify single-cell c-Fos expression within the VTA **(A)**, SNc **(C)**, and LC **(E)** following either l-VNS (left) or r-VNS (right). Sections were co-stained for tyrosine hydroxylase (red), c-Fos (green), and DAPI (blue). Arrow heads in enlarged insets show example cells classified as exclusively DAPI+ (cyan arrows); DAPI+, c-Fos+, and TH− (green arrows); DAPI+, c- Fos-, and TH+ (magenta arrows); or DAPI+, c-Fos+, and TH+ (white arrows). **(B,D,F)** In both VTA **(B)** and SNc **(D)**, the percentage of TH+ neurons did not differ between r-VNS and l-VNS treatment groups (top). However, the percentage of c-Fos+ cells (bottom) was significantly greater in the r-VNS group, in both TH+ and TH− cell populations. **(F)** In the LC, r-VNS self-administration resulted in a higher percentage of TH+ cells than l-VNS (top), but the percentage of TH+ and TH− cells that were found to be c-Fos+ did not differ between groups (bottom). In **(B,D,F)**, Student’s *t*-tests were used to test for between-group differences in TH+ population size, as well as in c-Fos expression within TH+ and TH− populations; multiple comparisons were corrected using false discovery rate. For FDR-corrected comparisons in which statistically significant differences were observed, uncorrected *p*-values are indicated: *p < 0.05, **p < 0.01, ***p < 0.001.

We next examined whether enhanced neuronal activity within the VTA, SNc, and LC occurred within the catecholaminergic or non-catecholaminergic cell populations in each nucleus. The nuclear marker DAPI was used to label cells in VTA, SNc, and LC and DAPI-labeled cells were classified as TH+ or TH−, as well as c-Fos+ or c-Fos- (Figures 3A,C,E). Within the VTA and SNc, we observed similar percentages of TH+ cells in r-VNS and l-VNS treated subjects (Figures 3B,D, Table 4, and Supplementary Table 1). However, compared to l-VNS treated rats, r-VNS treated subjects exhibited significantly greater c-Fos expression within both TH+ and TH− cell populations in these regions (Figures 3B,D, Table 4, and Supplementary Table 1). In VTA, r-VNS treated rats had approximately 4 times more c-Fos+ non-dopaminergic cells, and approximately 8 times more c-Fos+ dopaminergic neurons, than l-VNS treated rats. In SNc, r-VNS treated rats had ca. 2 times more c-Fos+ non-dopaminergic cells, and ca. 13 times more c-Fos+ dopaminergic neurons, than l-VNS treated rats. These findings indicate that r-VNS self-administration drives stronger midbrain neuronal activation than l-VNS, in both dopaminergic and non-dopaminergic populations within the VTA and SNc.

**TABLE 4:** Between-group comparisons of the percentage of TH+ cells, as well as percentage of c-Fos+ cells within the separate TH+ vs. TH− populations within VTA, SNc, and LC.

	% TH+ cells			% c-Fos+ of TH+ population			% c-Fos+ of TH− population		
	r-VNS	l-VNS	*t*-test	r-VNS	l-VNS	*t*-test	r-VNS	l-VNS	*t*-test
						
	Mean (SEM)		*p*-value (FDR *q*-value)	Mean (SEM)		*p*-value (FDR *q*-value)	Mean (SEM)		*p*-value (FDR *q*-value)
VTA	28.38 (0.98)	30.25 (0.63)	0.1856 (0.278)	51.74 (3.40)	6.26 (2.74)	**0.0005** (0.001)	13.00 (0.42)	3.35 (0.34)	**0.0001** (0.000)
SNc	20.52 (1.97)	24.74 (1.98)	0.2059 (0.265)	44.34 (2.10)	3.44 (1.54)	**0.0001** (0.000)	13.81 (0.52)	6.12 (1.33)	**0.0058** (0.013)
LC	51.32 (4.32)	31.39 (2.60)	**0.0167** (0.030)	77.45 (0.75)	60.86 (13.12)	0.2756 (0.310)	11.61 (2.96)	17.85 (5.85)	0.3952 (0.395)

*For each region and cell population, percentages were compared between r-VNS and l-VNS treatments using Student’s t-tests, corrected for multiple comparisons using false discovery rate. Bold denotes statistical significance for FDR-adjusted q-values < 0.05.*

In the noradrenergic LC, r-VNS self-administration was associated with a significant increase in TH+ staining compared to l-VNS treatment (Figure 3F, Table 4, and Supplementary Table 1). The overall percentage of c-Fos+ cells did not differ, however, between r-VNS and l-VNS treatment groups, for either TH+ or TH− cell populations in the LC. Within the TH+ noradrenergic population, the majority of neurons in both l-VNS and r-VNS treated rats were found to be c-Fos+, consistent with prior reports that VNS drives neural firing in the LC (Groves and Brown, 2005; Dorr and Debonnel, 2006; Hulsey et al., 2017) and enhances noradrenaline release throughout the brain (Dorr and Debonnel, 2006; Roosevelt et al., 2006; Follesa et al., 2007; Manta et al., 2009; Raedt et al., 2011). Our results further suggest that, compared to l-VNS, r-VNS self-administration results in greater overall activation of LC noradrenergic neurons, which may be accompanied by enhanced catecholamine synthesis within the LC.

## Discussion

In the current study, we tested whether VNS induces differential activation of midbrain dopaminergic nuclei when delivered to the right vs. left cervical vagus nerves. Our findings provide the first evidence, to our knowledge, that standard electrical stimulation of the right cervical vagus nerve is sufficient to reinforce learned behaviors, and to drive strong activation of midbrain dopaminergic neurons within the VTA and SNc. Notably, these effects were not observed with traditional l-VNS delivered at equivalent stimulation parameters. These results suggest that, compared to l-VNS, r-VNS can engage additional neuroplasticity-promoting signaling pathways, opening broad possibilities for further research into the therapeutic potential of r-VNS for the treatment of neurological disorders.

Our finding that r-VNS promotes appetitive behavioral responses while activating midbrain dopaminergic nuclei is consistent with recent literature detailing similar lateralization in the anatomical and functional connectivity between the upper gut and the midbrain DA system (Han et al., 2018). Using optogenetics to selectively target stomach and duodenum-innervating vagal cell bodies located in the left vs. right nodose ganglia (NG), Han et al. (2018) showed that activation of gut-innervating right, but not left, NG neurons drives striatal dopamine release and induces both place preference and increased nose poke behaviors. These functional effects were consistent with differential anatomical connectivity between left and right gut-innervating NG cells and brainstem and midbrain nuclei. In the current study, lateralization of VNS-driven reward-related signaling may be hypothesized to arise from activation of these lateralized gut-innervating vagal fibers characterized by Han et al. (2018) However, electrical stimulation should non-selectively activate vagal fibers innervating the upper gut in addition to those targeting the intestines, liver, pancreas and other organs capable of conveying reward-related nutritive or metabolic information to the brain (Yuan and Silberstein, 2016; Browning et al., 2017; Shechter and Schwartz, 2018; Berthoud and Neuhuber, 2019; de Araujo et al., 2020). Indeed, other authors have recently reported an increase in VTA activation following optogenetic stimulation of left nodose ganglion neurons (Fernandes et al., 2020). Together, these findings suggest that additional studies are needed to fully specify the peripheral origins of lateralized VNS effects.

The acclimation protocol used here presents an important limitation of our study. In the current experiments, we find evidence of strong lateralization in VNS-driven reward-related signaling in rats that had been previously trained to lever press for food reward. A recent study by Fernandes et al. (2020) provides some evidence that this food-reinforced training period may impact appetitive vagal-mesencephalic connectivity. These authors found that intragastric infusions of a sucrose solution were strongly reinforcing in mice previously trained to lever-press for oral sucrose delivery, but that the same infusions were not rewarding in naïve mice. Their findings are consistent with prior reports that significant plasticity occurs within reward-related gut-brain signaling pathways following orogastric reward consumption (Berthoud, 2008; Uematsu et al., 2009; Myers et al., 2013; Schier and Spector, 2016; Shechter and Schwartz, 2018; Bai et al., 2019; de Araujo et al., 2020). Further research is necessary to clarify the impact of such plasticity on r-VNS driven dopamine signaling.

Traditional l-VNS has been shown to enhance several learning and memory processes. Preclinically, l-VNS is seen, for example, to induce significant neuroplasticity within the motor system (Porter et al., 2012; Morrison et al., 2019; Tseng et al., 2020), and to improve functional recovery following stroke and other neural injuries (Hays et al., 2014; Pruitt et al., 2016; Meyers et al., 2018, 2019). Left VNS has also been found to speed extinction and prevent reinstatement of drug seeking (Childs et al., 2017, 2019) and of conditioned fear (Peña et al., 2013, 2014; Childs et al., 2015; Burger et al., 2016; Noble et al., 2017, 2019; Szeska et al., 2020; Souza et al., 2021). These effects of l-VNS are thought to depend on the coordinated activation of multiple neuromodulatory systems, including broadly projecting cholinergic, noradrenergic, and serotonergic systems (Detari et al., 1983; Krahl et al., 1998; Dorr and Debonnel, 2006; Osharina et al., 2006; Cunningham et al., 2008; Manta et al., 2009; Ruffoli et al., 2011; Hays, 2016; Hulsey et al., 2016, 2019; Meyers et al., 2019; Collins et al., 2021). Consistent with the results of the current study, we recently demonstrated that cortical dopamine is not required for l-VNS induced neuroplasticity to occur (Brougher et al., 2021), nor is l-VNS typically found to be inherently rewarding (Noble et al., 2019; Hickman et al., 2021; Müller et al., 2021). Taken together with this prior literature, the results of the current study thus suggest that, unlike l-VNS, r-VNS strongly engages the midbrain dopaminergic system. Given the strong dependence on multiple neuromodulatory signaling pathways, it is perhaps unsurprising that l-VNS efficacy exhibits an inverted U-shaped curve, with maximum efficacy in rats occurring at the parameters similar to those used in the current study, and reduced effectiveness occurring at lower and higher intensities of stimulation (Borland et al., 2016; Buell et al., 2018; Morrison et al., 2019; Pruitt et al., 2020; Souza et al., 2021). Dopaminergic signaling is known to exert similar inverted U-shaped effects on working memory, attention, and impulsivity (Williams and Dayan, 2005; Gjedde et al., 2010; Cools and D’Esposito, 2011). The parametric responses of midbrain dopaminergic activity to l-VNS and r-VNS have not been well characterized, but understanding this relationship will be critical for optimizing the therapeutic potential of targeted vagal-mesencephalic stimulation.

Importantly, l-VNS is specifically approved for clinical use due to concerns that stimulation of the right nerve may be more likely to induce adverse cardiac effects. This concern largely arises from the anatomical observation that the sinoatrial node is preferentially innervated by right vagus fibers, while the cardiac ventricles receive innervation from both right and left nerves (Krahl, 2012; Coote, 2013). However, evidence that more severe cardiac effects are produced by r-VNS is mixed and varies according to the model species used (Ardell and Randall, 1986; Lockard et al., 1990; Lewis et al., 2001; Krahl et al., 2003). Due to its hypothesized cardiac effects, right cervical VNS is now under investigation for the treatment of heart failure (Zannad et al., 2015; Gold et al., 2016; Anand et al., 2020; Hadaya and Ardell, 2020). While r-VNS has been safe and well-tolerated in these trials, it was found to be ineffective (Zannad et al., 2015; Gold et al., 2016), or no more effective than l-VNS (Premchand et al., 2014, 2016; Nearing et al., 2021), at improving cardiac function. Moreover, in several clinical case reports (McGregor et al., 2005; Spuck et al., 2008), as well as in preclinical studies (Krahl et al., 2003; Sun et al., 2012), r-VNS was seen to improve neurological symptoms, without inducing severe adverse effects. While significantly more data are needed, existing evidence indicates that r-VNS may be safe and well-tolerated for neurological indications. Our current findings suggest that targeting the right cervical nerve rather than the left could potentially enhance the therapeutic efficacy of VNS for indications in which dopamine signaling is known to be disrupted, including, for example, Parkinson’s disease, major depressive disorder, or obesity.

## Data Availability Statement

The raw data supporting the conclusions of this article will be made available by the authors, without undue reservation.

## Ethics Statement

The animal study was reviewed and approved by the Institutional Animal Care and Use Committee at the University of Texas at Dallas.

## Author Contributions

JB and CT conceived the experiments, analyzed the data, and wrote the manuscript. JB, UA, NA, MC, AJ, IS, and SS performed the experiments and histological quantification. All authors contributed to the manuscript and approved of the final submission.

## Conflict of Interest

The authors declare that the research was conducted in the absence of any commercial or financial relationships that could be construed as a potential conflict of interest.

## Publisher’s Note

All claims expressed in this article are solely those of the authors and do not necessarily represent those of their affiliated organizations, or those of the publisher, the editors and the reviewers. Any product that may be evaluated in this article, or claim that may be made by its manufacturer, is not guaranteed or endorsed by the publisher.
