# Supplementary material for: Self-Administration of Right Vagus Nerve Stimulation Activates Midbrain Dopaminergic Nuclei
Source: Front Neurosci. 2021 Dec 16;15:782786. doi: 10.3389/fnins.2021.782786 (PMC8716493; doi:10.3389/fnins.2021.782786)
Supplement: Supplementary file 1 [file Image_1.pdf]

Supplementary Material

Self-administration of right vagus nerve stimulation activates midbrain dopaminergic nuclei

Jackson Brougher, Umaymah Aziz, Nikitha Adari, Muskaan Chaturvedi, Aryela Jules, Iqra Shah, Saba Syed, Catherine A. Thorn*

Department of Neuroscience, University of Texas at Dallas, Richardson, Texas, USA

*** Correspondence:**
Catherine Thorn
catherine.thorn@utdallas.edu

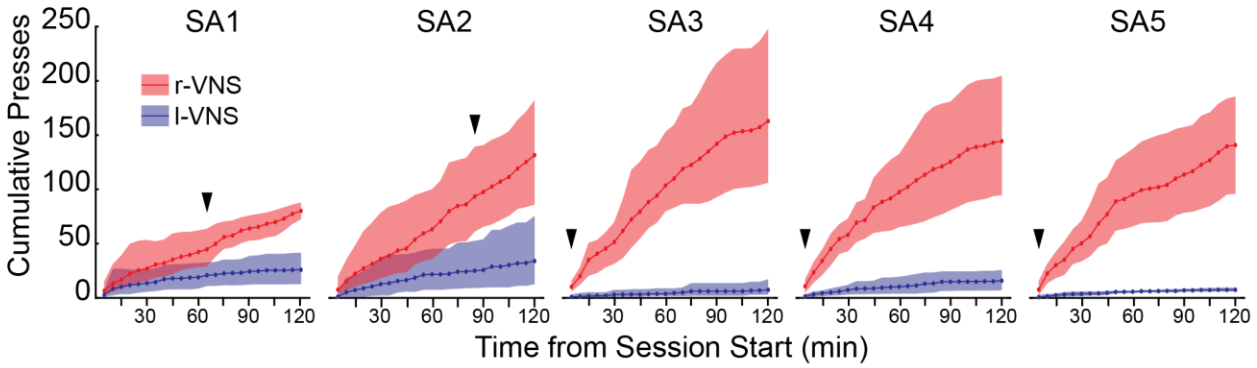

Supplementary Figure 1. Rats self-administer r-VNS but not l-VNS. Data show mean cumulative lever presses and bootstrapped 95% confidence intervals (1000 bootstraps) for r-VNS (red) and l-VNS (blue) treatment groups in 5-minute intervals over each 2-hour VNS-SA session. Within each of the 5 VNS-SA sessions, rats receiving r-VNS performed significantly more total lever presses than rats that received l-VNS. Arrowheads denote the first bin in each session in which a statistically significant between-group difference in cumulative presses was observed; within each session, multiple between-group (unpaired) t-test comparisons were corrected using false discovery rate. During the first VNS-SA session (SA1), differences in cumulative lever pressing began to reach statistical significance after 65 minutes. In SA2, significant differences were observed after 85 minutes. In SA3-SA5, significant between-group differences in lever pressing were seen within the first 5 minutes of each session.
